# Transformation and biodegradation of 1,2,3-trichloropropane (TCP)

**DOI:** 10.1007/s11356-012-0859-3

**Published:** 2012-08-08

**Authors:** Ghufrana Samin, Dick B. Janssen

**Affiliations:** Department of Biochemistry, Groningen Biomolecular Sciences and Biotechnology Institute, University of Groningen, Nijenborgh 4, 9747 AG Groningen, the Netherlands

**Keywords:** 1,2,3-Trichloropropane, Biodegradation, Bioremediation, Chlorinated hydrocarbons, Dehalogenase

## Abstract

**Purpose:**

1,2,3-Trichloropropane (TCP) is a persistent groundwater pollutant and a suspected human carcinogen. It is also is an industrial chemical waste that has been formed in large amounts during epichlorohydrin manufacture. In view of the spread of TCP via groundwater and its toxicity, there is a need for cheap and efficient technologies for the cleanup of TCP-contaminated sites. In situ or on-site bioremediation of TCP is an option if biodegradation can be achieved and stimulated. This paper presents an overview of methods for the remediation of TCP-contaminated water with an emphasis on the possibilities of biodegradation.

**Conclusions:**

Although TCP is a xenobiotic chlorinated compound of high chemical stability, a number of abiotic and biotic conversions have been demonstrated, including abiotic oxidative conversion in the presence of a strong oxidant and reductive conversion by zero-valent zinc. Biotransformations that have been observed include reductive dechlorination, monooxygenase-mediated cometabolism, and enzymatic hydrolysis. No natural organisms are known that can use TCP as a carbon source for growth under aerobic conditions, but anaerobically TCP may serve as electron acceptor. The application of biodegradation is hindered by low degradation rates and incomplete mineralization. Protein engineering and genetic modification can be used to obtain microorganisms with enhanced TCP degradation potential.

## Introduction

1,2,3-Trichloropropane (TCP, CAS No. 96-18-4) is a non-natural, biodegradation-recalcitrant and toxic compound that occurs in groundwater and soil, mainly as a result of improper disposal of TCP-contaminated chemical waste (Agency for Toxic Substances and Disease Registry [ATSDR] 1992, 2011). TCP is formed as a by-product during the synthesis of various chemicals, most notably in the classical synthetic route to epichlorohydrin, and was present in commercial preparations of the soil fumigant 1,3-dichloropropene (also known under the trade name D-D), which is now abandoned (Tesoriero et al. 2001). TCP is also applied as an intermediate in the production of various other chemicals. For example, fluorination of TCP is used to produce the cross-linking agent hexafluoropropylene, which is applied for making elastomers. TCP is also used in the chemical industry as a solvent for oils and fats, waxes, and resins. Other past uses of this compound include in paint thinner and varnish remover, and as a degreasing agent. The Toxic Substances Control Act inventory of the US Environmental Protection Agency (US EPA) estimated the usage of TCP in 2002 as 1–5 × 10^6^ kg (US EPA 2004). The Federal Facilities Restoration and Reuse Office (FFRRO) of the US EPA has listed TCP as an Emerging Contaminant in December 2010 for which physical, toxicological and environmental data were summarized in a fact sheet (FFRRO 2011) and a review of the toxicology of TCP was written (US EPA 2009). The European Union Chemicals Agency (ECHA) has listed TCP as a chemical of very high concern because of its carcinogenic, mutagenic and reproductive effects (ECHA 2012a).

Contamination of soil and groundwater by TCP has occurred both as point source pollution, due to improper disposal of wastes or accidental spillage, and as diffuse contamination, due to its presence in the nematicide 1,3-dichloropropene. TCP has been detected in hundreds of surface water and drinking water sources, e.g., in the United States, at levels of 0.1–100 μg/l (Oki and Giambelluca 1989; ATSDR 1992, 2011; California Environmental Protection Agency 2009). In a Dutch monitoring program, TCP was detected in surface water of the Rhine, Meuse, Westerscheldt, and in the Northern Delta Area (Miermans et al. 2000). Groundwater samples from the Netherlands were found to contain TCP as well as 1,2-dichloropropane due to the application of impure nematicides, especially in potato fields (Lagas et al. 1989). TCP was also detected in the river Nitra, Slovakia (Liska et al. 1996) and along with a range of other volatile organohalogens in water at an industrial site in the Osaka area, Japan (Yamamoto et al. 1997). These examples illustrate that TCP is a very widespread contaminant.

TCP is a suspected human carcinogen based on evidence of tumor formation in studies with rats and mice (Irwin et al. 1995; US EPA 2009). Because of its toxicity, the presence of TCP in groundwater can pose a serious risk to human health and ecosystem quality, and the National Toxicology Program (1993, 2005) of the US Department of Health has listed TCP as reasonably anticipated to be a human carcinogen (US National Library of Medicine 2011). Toxicological properties of TCP are included in the ECHA Registered Substances Database (ECHA 2012b) and the NIH Hazardous Substances Data Bank (HSDB 2000).

Remediation of TCP-contaminated sites is difficult due to its persistent nature and its physiochemical properties, which cause spreading with flowing groundwater (Salter et al. 2010). Biodegradation has been observed both under anaerobic and aerobic conditions, but appears to be slow and mostly due to cometabolism, with little evidence for TCP supporting growth or adaptation of bacteria. Based on laboratory studies there are indications that TCP may serve as an electron acceptor under anaerobic conditions. In this review, we discuss abiotic and biotic transformations of TCP, as well as the possibilities of enzymatic dehalogenation of TCP and genetic construction of TCP-degrading bacteria.

## TCP as an environmental chemical

TCP, also known as allyl trichloride, trichlorohydrin or glycerol trichlorohydrin, is a clear and colorless liquid, with a strong odor similar to that of chloroform or trichloroethylene. It is soluble in ethanol, ether, and chloroform, and only slightly soluble in water. Like other chlorinated hydrocarbons, it reacts with some metals, strong basic agents, and oxidizing agents. It is sensitive to prolonged exposure to light and heat. TCP is flammable, and when heated to decomposition, it yields toxic fumes of hydrogen chloride gas (US National Library of Medicine 2011).

### Metabolism and toxicity of TCP

**Fig. 1:**
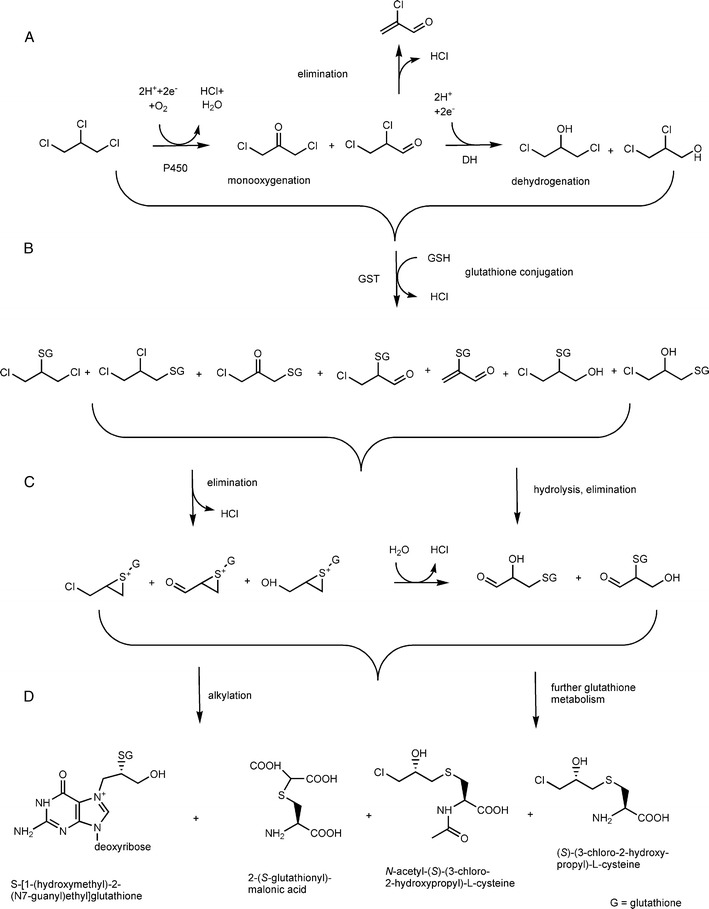
Metabolic pathways of 1,2,3-trichloropropane (TCP) (Mahmood et al. 1991; La et al. 1995; WHO 2003; US EPA 2009). Reactions: **a** initial monooxygenase (P450)-mediated conversion and dehydrogenation leading to dichloro-metabolites; **b** glutathione conjugation can occur on TCP and oxidized derivatives; **c** glutathione adducts can be converted to highly toxic species, such as episulfonium ions; **d** the final products include DNA adducts, as well as glutathione derivatives that are secreted

The toxicological properties of TCP were recently reviewed by the US EPA (2009) and earlier by the WHO (2003).The major pathways for metabolism of TCP in higher organism starts with oxidative transformation by microsomal cytochrome P-450 or substitution by glutathione transferase (Mahmood et al. 1991; Weber and Sipes 1992). P450-monooxygenase-mediated conversion leads to formation of 2,3-dichloropropanal and 1,3-dichloroacetone, which can be reduced to 2,3-dichloropropanol and 1,3-dichloropropanol, presumably by dehydrogenase activity (Fig. 1). 2,3-Dichloropropanal also decomposes non-enzymatically to 2-chloroacrolein. Glutathione conjugation of TCP may produce glutathione adducts that can undergo intermolecular substitution to form a highly reactive episulfonium ion, but glutathione can also prevent alkylation of proteins by the electrophilic monooxygenase products 2,3-dichloropropanal and 1,3-dichloroacetone (Weber and Sipes 1992). Metabolism of glutathione conjugates formed from TCP and its oxidation products also gives rise to various glutathione, cysteine and *N*-acetylcysteine conjugates. Thus, TCP itself is not mutagenic, but the products (such as 2-chloroacrolein and episulfonium ions) are strong alkylating agents, explaining the toxicity and mutagenicity of TCP. Using radiolabeled TCP, formation of DNA adducts after exposure to TCP has indeed been demonstrated in mice (La et al. 1995).

Animal studies have shown that long-term exposure to TCP may cause kidney failure, reduced body weight and tumors (Irwin et al. 1995; ATSDR 1992; US EPA 2009; ECHA 2012b). The guidelines for carcinogen risk assessment and IRAC monographs reported that TCP is expected to be carcinogenic to humans, based on animal studies, as well as resemblance of metabolism between human and rodent microsomes, in vitro mutagenicity, and the ability to form DNA adducts (Irwin et al. 1995; Ya et al. 1996; US EPA 2009).

### Environmental fate of TCP

Properties that determine the behavior of TCP in the environment include: its high density (1.39 g/ml); the modest water solubility (1.75 g/l at 20 °C; for trichloroethylene the value is 1.4 g/l at 20 °C); the low octanol–water partitioning coefficient (log *K*_ow_ = 2.0–2.5, quite similar to the value for trichloroethylene); and the low Henry coefficient (*H* = 3.2–3.4 × 10^-4^ atm m^3^/mol at 25 °C). The high density causes TCP, when dumped as a liquid on soil or in ponds, to sink to lower levels, e.g., into groundwater or into accessible subsurface structures like rock fissures. The low octanol–water partitioning coefficient implies substantial distribution via groundwater flows, even when organic carbon is present. Furthermore, TCP has a tendency to evaporate from surface water to air (but less than trichloroethylene, *H* = 10 × 10^−4^ atm m^3^/mol), where, on exposure to sunlight, it is subjected to photo-degradation by reaction with hydroxyl radicals with a half-life of about 15 days. The volatility of TCP, the possibility of washout by precipitation, and its resistance to degradation in water, may result in cycling of TCP between environmental compartments (ATSDR 1992; US EPA 2009). Bioaccumulation and biomagnification are expected to be of minor importance in view of the modest lipophilicity of TCP.

**Fig. 2:**
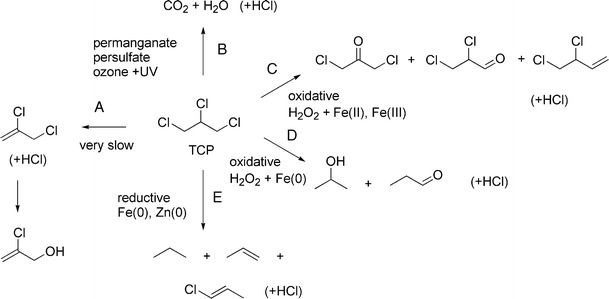
Abiotic transformations of TCP under non-stimulated conditions (**a**), photochemical conversion or catalytic conversion in the presence of radical-generating oxidants (**b**), conversion in the presence of Fenton reagent (**c**, **d**), and conversion under stimulated anaerobic (reductive) conditions (**e**). Non-catalyzed conversion under neutral conditions is very slow. See text for details

TCP can stay in groundwater for a prolonged period, in part due to its low organic carbon partitioning coefficient, but especially because rates of abiotic and biotic degradation in groundwater are low. Abiotic hydrolysis of TCP under basic and neutral abiotic conditions has been studied at different temperatures and pH values and in the presence of different ions such as sulfide and carbonate (Sarathy et al. 2010). TCP appears to be highly stable under a variety of conditions, with an expected half-life of hydrolysis under environmental conditions (25 °C, pH = 7) in the order of hundreds of years (Pagan et al. 1998). At high temperatures, 2,3-dichloro-1-propene was detected as a product that could be converted to 2-chloro-2-propen-1-ol (Fig. 2). Other non-stimulated abiotic reactions in water under environmental conditions have not been characterized.

### Remediation of TCP-contaminated sites: abiotic transformations

The physical and chemical properties of TCP obviously strongly influence possibilities for remediation of soil and groundwater (Salter-Blanc and Tratnyek 2011). On-site methods for treating TCP-polluted groundwater include pump and treat and vacuum extraction. The latter is used for different volatile organohalogens, but it is not a very favorable approach because of the low Henry’s law constant of TCP (CH2M HILL 2005). Extracted water can be treated by absorption, using activated carbon, by chemical oxidation or by vacuum extraction. For example, full-scale remediation of TCP-contaminated groundwater is under investigation at the Tyson superfund site near Philadelphia, PA, using vacuum extraction of soil, extraction of groundwater, and treatment of extracted water and vapor with activated carbon for the removal of TCP (Pezullo et al. 2005).

Chemical oxidation is performed with oxidizing agents such as ozone, permanganate or hydrogen peroxide. They cause decomposition of TCP into carbon dioxide, water and chloride ions (Dombeck and Brog 2005) (Fig. 2). With a mild oxidant such as permanganate, transformation of TCP is slow whereas in the presence of strong oxidants such as hydroxyl radicals, generated photochemically, and sulfate radicals, catalytically generated from persulfate, transformation is much faster (CH2M HILL 2005).

Chemical TCP oxidation may also be initiated by the Fenton reagent (H_2_O_2_ with an iron catalyst). Khan et al. (2009) studied the effect of iron type (Fe^+2^, Fe^+3^ and Fe^0^) on the removal of TCP by H_2_O_2_, and found that Fe^2+^ was most effective in reducing TCP and increasing its biodegradability. Degradation products were 1,3-dichloroacetone and 2,3-dichloro-1-propene if Fe^2+^ and Fe^3+^ were used, and isopropanol and propionaldehyde if Fe^0^ was used, confirming extensive oxidative conversion. Such oxidative transformation studies have been done in batch reactors. Chemical oxidation can also be used for in situ treatment of TCP contamination. In case TCP is present in the form of a dense non-aqueous-phase liquid (DNAPL), oxidants can be introduced into the subsurface to achieve contaminant oxidation.

Chlorinated solvents such as trichloroethene, carbon tetrachloride and TCP may also be removed by abiotic reduction with zero-valent iron. Thus, distribution of TCP via flowing groundwater can be prevented by using a zero-valent iron (Fe^0^) barrier. The predominant removal mechanisms are sorption and reductive abiotic transformation. Klausen et al. (2003) investigated the effects of carbonate, silica, chloride and organic matter on the removal of various organohalides by granular iron using column studies. The results indicated that differences in groundwater chemistry have a strong effect on the activity and longevity of the granular iron, which will influence the design of reactive barriers. Compounds enhancing metal corrosion (carbonate, chloride) may improve reactivity, whereas compounds such as FeCO_3_ and Na_2_SiO_3_ can reduce the activity, especially upon prolonged treatment, through deactivation of the metal surface. Propane, propylene, and trace amounts of 1-chloro-2-propene were detected as TCP transformation products (Fig. 2), indicating a role for reductive dechlorination and elimination of HCl in the removal of TCP.

Reductive transformation of TCP was also found with zero-valent zinc, which exhibited a reactivity that was more than an order of magnitude higher than that of iron (Sarathy et al. 2010; Salter-Blanc and Tratnyek 2011). Groundwater components that influenced zinc surface properties through corrosion or formation of an inactive layer of ZnO or Zn(OH)_2_ had a large influence on the removal kinetics. No degradation products other than propene were detected, suggesting that dechlorination is extensive.

## Biotransformation and biodegradation

### Anaerobic biodegradation of TCP

Few studies have been done aimed at establishing the possibilities for biodegradation and bioremediation of TCP. Growth-supporting biodegradation of halogenated compounds is generally based on one of the following processes: (1) chemotrophy with an oxidizable electron donor (hydrogen, lactate) and use of the halogenated compounds as a physiological electron acceptor (anaerobic conditions); (2) use of the halogenated compound as a carbon and energy source with an external electron acceptor (oxygen, nitrate); (3) fermentative metabolism, in which the halogenated compound serves both as electron donor and (indirectly) as electron acceptor. Biotransformation processes not linked to growth may also be important. Such cometabolic transformations are due to the broad substrate spectrum of many microbial enzymes, the general reactivity of cofactors, or the formation of reactive intermediates in the catalytic cycle of some enzymes. Examples are reductive dechlorination by cobalamin cofactors of anaerobic bacteria, and oxidative transformation by broad-specificity metal-containing monooxygenases of aerobic bacteria. Obviously, a biodegradation process that stimulates growth of the active organisms is preferable in a bioremediation situation since it allows adaptation at the population level, leading to an increase of the amount of active biomass during the treatment process.

**Fig. 3:**
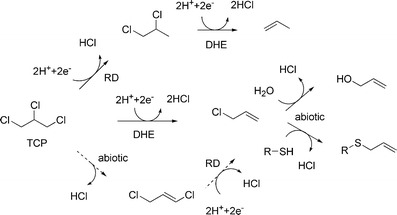
Anaerobic biotransformations of TCP. Both reductive dehalogenation (*RD*) and dihaloelimination reactions (*DHE*) are observed. Formation of allylchloride may occur by dihaloelimination (Yan et al. 2009) or possibly via 1,3-dichloropropene (*broken arrows*)

Reductive biotic transformation of TCP has been demonstrated under anaerobic conditions (Löffler et al. 1997; Peijnenburg et al. 1998; Hauck and Hegemann 2000) (Fig. 3) and sequential aerobic–anaerobic conditions (Long et al. 1993). Reductive dechlorination of both TCP and chloroethanes was observed with an enrichment culture that dechlorinated 1,2-dichloropropane, and propene and 1,2-dichloropropane were detected as products (Löffler et al. 1997). Experiments in which various halogenated aliphatic compounds were incubated with anaerobic sediments indicated zero-order conversion kinetics for TCP and dichloromethane, whereas most other organohalogens were transformed according to first order kinetics (Peijnenburg et al. 1998).

Recently, two strains (BL-DC-8 and BL-DC-9) of an anaerobic Gram-negative bacterium were isolated from contaminated groundwater at a Superfund site located near Baton Rouge, and characterized as belonging to the new species *Dehalogenimonas lykanthroporepellens* (Moe et al. 2009; Yan et al. 2009). These bacteria utilize TCP as an electron acceptor under anaerobic conditions but not chlorinated alkenes. Hydrogen was the electron donor. For both strains, allyl chloride was detected as the main initial dechlorination product (Fig. 3). However, allyl chloride is unstable and is hydrolyzed abiotically to allyl alcohol, whereas in the presence of cysteine or sulfide, allyl chloride was transformed to allyl mercaptan, *S*-allyl mercaptocysteine and allyl sulfides. The mechanism of the reductive dechlorination reaction is not completely clear, as the enzymes responsible for TCP dechlorination have not yet been isolated and characterized. In freshwater environments, transformation of TCP into allyl chloride followed by the formation of allyl alcohol could be toxic to fish and aquatic life (Ewell et al. 1986).

Bioremediation of contaminated groundwater through in situ reductive dechlorination can be performed by injecting a compound such as hydrogen, lactic acid or another oxidizable organic substrate that is used by microorganisms to produce hydrogen, which induces reductive dechlorination and serves as electron donor (Tratnyek 2008). At a site in California, 99.9 % reduction of TCP contamination has been found over a period of 1,000 days. However, biotic dechlorination through hydrogen-releasing compounds may be applicable only at low concentrations, such as less than 1 mg TCP/l (CH2M HILL 2005).

### Aerobic cometabolic conversion

**Fig. 4:**
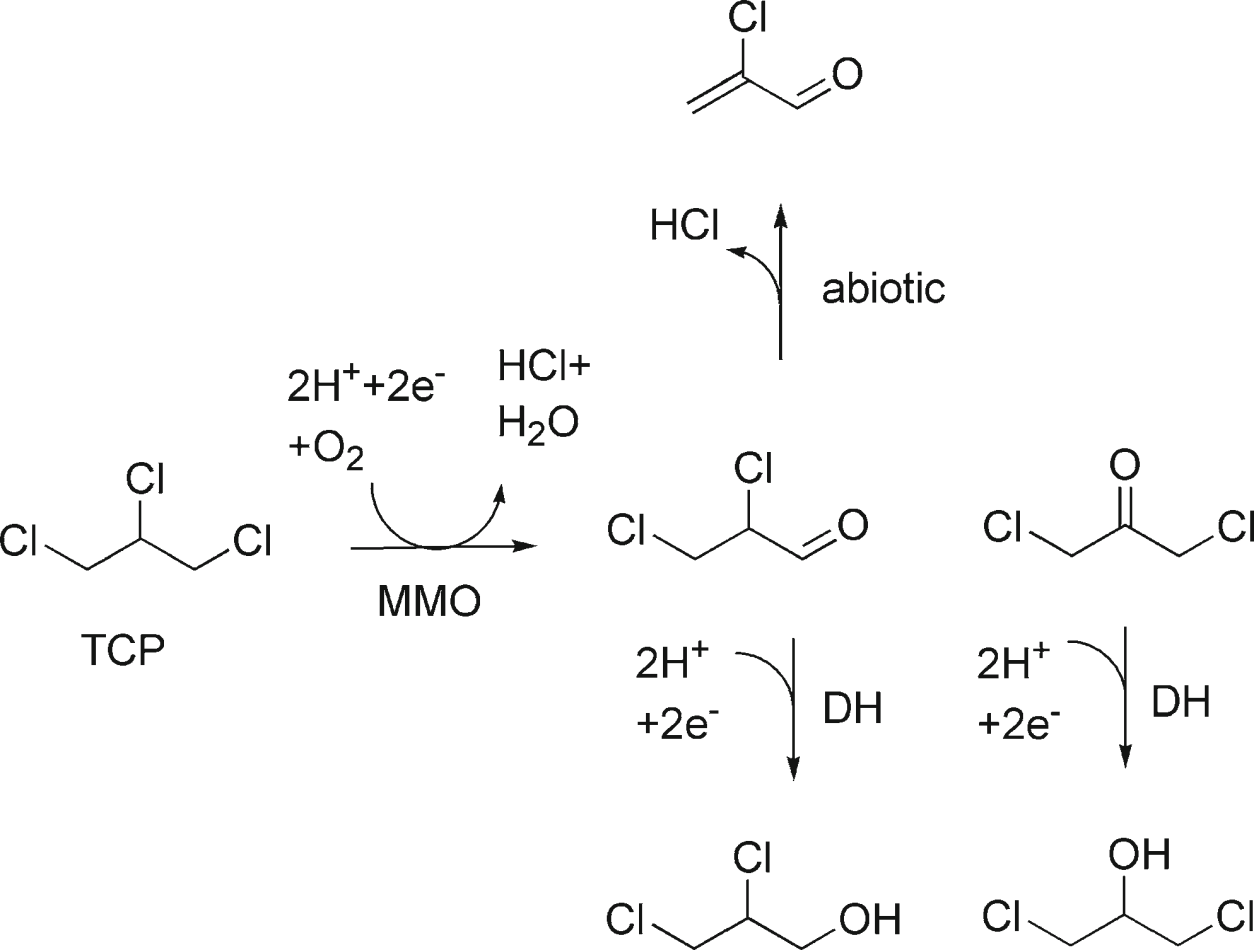
Conversions of 1,2,3-trichloropropane initiated by methane monooxygenase (*MMO*) produced by *M. trichosporium* OB3b cells. Reduction to alcohols is caused by alcohol dehydrogenase activity (*DH*)

Various halogenated aliphatic hydrocarbons can be transformed in a cometabolic manner by broad-specificity monooxygenase involved in hydrocarbon degradation, such as methane monooxygenase (Hanson et al. 1990; Oldenhuis et al. 1989). The soluble methane monooxygenase produced by cells of the methanotrophic bacterium *Methylosinus trichosporium* OB3b can convert TCP, giving rise to dichloropropanols after subsequent reduction (Bosma and Janssen 1998; Fig. 4). However, TCP is a poor substrate for the enzyme as compared to other pollutants such as trichloroethylene. The conversions are analogous to those catalyzed by cytochrome P450 in mammalian systems (Fig. 1). The major drawback of such cometabolic conversions is product toxicity. In case of TCP conversion by methane monooxygenase, the insertion of oxygen preferentially occurs on the terminal carbon atom, which yields chlorinated carbonyl compounds that may undergo elimination to produce 2-chloroacrolein, a very reactive compound.

The aerobic conversion of TCP reported by Leahy et al. (2003) using a mixture of hydrocarbon-degrading bacteria is probably based on similar reactions, but the products were not identified. Aromatic hydrocarbon-degrading bacteria produce monooxygenases that are capable of chlorinated hydrocarbon degradation through similar oxidative reactions as the methane monooxygenase of methanotrophs. For example, the toluene monooxygenase of a pseudomonad was described to convert chlorinated hydrocarbons (Newman and Wackett 1997).

### Recalcitrant behavior of TCP towards growth-supporting aerobic biotransformation

Microbial transformation of TCP to CO_2_, H_2_O and HCl by oxidative metabolism with oxygen as an electron acceptor and by reduction to lesser chlorinated propanes and HCl is thermodynamically possible (Dolfing and Janssen 1994). However, no aerobic organisms, enrichment cultures, or bioreactors have been described that demonstrate the use of TCP as a growth-supporting oxidizable substrate. Various attempts to enrich TCP-degrading microorganisms from environmental samples, including from sites with a long history of TCP or epichlorohydrin pollution, or to obtain TCP degradation in continuous-flow columns inoculated with samples from contaminated sites, have failed (Bosma et al. 2002). This indicated that TCP is indeed a very recalcitrant compound and nature has not yet evolved aerobic organism that are adapted to it. The fact that the thermodynamic calculations indicate that aerobic oxidation of TCP is energetically favorable suggests biochemical hurdles instead of another fundamental reason as the cause of the apparent recalcitrance of TCP.

An example of such a biochemical hurdle is toxicity of intermediates. In case of halogenated aliphatic compounds, several reactive intermediates occur along the metabolic pathways, requiring optimization of fluxes to prevent accumulation of such reactive intermediates to toxic levels (van Hylckama Vlieg and Janssen 2001). It also may be due to formation of dead-end side products that are toxic. Formation of such reactive intermediates will act against evolutionary selection of more efficient initial enzymes for TCP metabolism. The recalcitrant nature of a non-natural compound might also be due to presence of structural elements that cannot be recognized and converted by microbial enzymes, which evolved for the conversion of natural compounds (Rieger et al. 2002).

When inspecting the possible pathways for productive aerobic metabolism of TCP, hydrolysis of a carbon–halogen bond as the first step seems the most attractive reaction, because it does not involve reactive intermediates and leads to 1,3-dichloro-2-propanol 2,3-dichloro-1-propanol. These compounds are known to be biodegradable and pure cultures capable of using dichloropropanols for growth under aerobic conditions are known (Effendi et al. 2000; Higgins et al. 2005; van den Wijngaard et al. 1989; Yonetani et al. 2004).

Hydrolysis of carbon–halogen bonds in chlorinated compounds is carried out by a diversity of microbial enzymes called dehalogenases. These belong to different phylogenetic classes, of which the haloalkane dehalogenases that are members of the α/β-hydrolase fold superfamily of proteins are the best characterized (Janssen 2004; Koudelakova et al. 2011). Another prominent class is the HAD-superfamily of haloacid dehalogenases and phosphatases, with dehalogenases that act on 2-chloroacetate and 2-chloropropionate. Haloalkane dehalogenases are known to convert compounds such as 1,2-dichloroethane, 1,2-dibromoethane, 1,3-dichloropropane, 1,2-dichloropropene, and (slowly) hexachlorocyclohexane (Janssen et al. 2005; Poelarends et al. 1999, 2000; Koudelakova et al. 2011). The conversion of TCP by a haloalkane dehalogenase was first described by Yokota et al. (1987) using an enzyme from *Corynebacterium* strain m15-3, but the activity was very low *(k*_cat_/*K*_m_ = 36 s^−1^ M^−1^ *)* (Bosma et al. 1999). Sequence analysis and structural studies identified the protein (which is commonly called DhaA) as a member of the α/β-hydrolase fold family. Another dehalogenase that has a low activity with TCP is LinB, and enzyme originally discovered in bacteria that degrade hexachlorocyclohexane (Monincová et al. 2007).

The first DhaA gene sequence was described by Kulakova et al. (1997) in the 1-chlorobutane degrader *Rhodococcus rhodochrous* NCIMB13064. Poelarends et al. (2000) found that the same gene is geographically widely distributed by using PCR analysis and dehalogenase gene sequencing of different bacteria enriched with other haloalkanes, including 1,3-dichloropropene. Comparison of the genetic organization in different organisms revealed that the haloalkane dehalogenase gene likely originates from *Rhodococcus* strains, where it is present in an operon together with an alcohol dehydrogenase and an aldehyde dehydrogenase gene, as well as a regulatory gene that influences gene expression. The latter may act as a repressor in the absence of a halocarbon substrate (like 1-chlorobutane). When the dehalogenase gene regions from a 1,2-dibromoethane degrading *Mycobacterium* and a 1,3-dichloropropene dehalogenating *Pseudomonas* were examined, it appeared that the repressor gene was absent or inactivated by mutations to allow production of the enzyme in the presence of these new, non-inducing substrates (Poelarends et al. 1999, 2000). In the absence of a functional regulatory gene, inactivation of the repressor causing constitutive expression of a dehalogenase appears a way to allow genetic adaptation and biodegradation.

Lack of microbial growth on TCP and lack of adaptation in column or enrichment experiments is most likely due to the very rare occurrence of a haloalkane dehalogenase gene with a suitable activity in a host organism that is capable of dichloropropanol conversion. Mutations in the haloalkane dehalogenase that would lead to an enhanced substrate range that includes TCP would be unlikely to propagate in an organism that does not grow on the hydrolysis product and thereby provide a selective growth advantage. When DNA sequence databases, both of completed bacterial genomes and environmental sequences, are searched for genes that encode the DhaA-type haloalkane dehalogenase, or the haloalcohol dehalogenases known to be involved in 2,3-dichloro-1-propanol metabolism (except in organisms isolated on these compounds), no hits are found. These genes seem very rare and can only be recovered by appropriate enrichment culture techniques starting with polluted environmental samples.

**Fig. 5:**
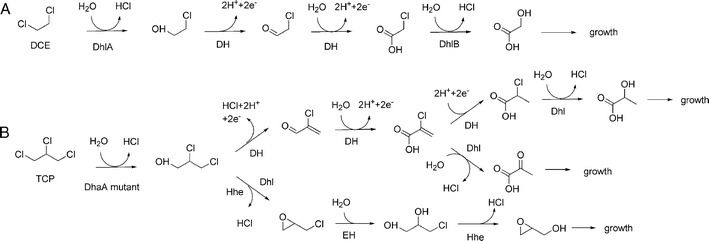
Comparison of catabolic pathways for 1,2-dichloroethane (*DCE*) and TCP. DCE bioremediation has been established at full scale, using bacterial cultures that use DCE as carbon source for growth according to the pathway that is shown (**a**). It starts with hydrolytic dehalogenation catalyzed by a haloalkane dehalogenase (*DhaA*). TCP is much more recalcitrant, but productive catabolic pathways can be envisaged (**b**). The upper routes could proceed from 2-chloroacrylic acid either via dehydrogenation (*DH*) (Kurata et al. 2005) or dechlorination (*Dhl*) (Mowafy et al. 2010). The lower route is thought to proceed in the strain constructed by Bosma et al. (2002) in *A. radiobacter* AD1 expressing a mutants haloalkane dehalogenase (*DhaAM2*) and involves dehalogenases (*Hhe*) and epoxide hydrolase (*EH*)

The evolution of bacteria that have the capacity to degrade TCP aerobically is thus restricted by the selectivity of haloalkane dehalogenases, and the rare occurrence of bacteria growing on dichloropropanols (Fig. 5). Consequently, attempts were made to obtain organisms capable of TCP detoxification by a combination of protein engineering and heterologous gene expression (Bosma et al. 1999, 2002).

### Engineering enzymes and organisms for TCP conversion

Different reports on the engineering of haloalkane dehalogenase variants with enhanced activity towards TCP have been published. By using error prone PCR and DNA shuffling, Bosma et al. (2002) generated a DhaA mutant (e.g., a variant called DhaAM2 with the mutations C176Y and Y273F) that had three times higher catalytic efficiency (*k*_cat_/*K*_m_ *=* 280 s^−1^ M^−1^) than wild-type enzyme. Similarly, Gray et al. (2001) performed in vitro evolution studies which also yielded a mutant with a substitution at position 176 and a mutation close to the N terminus that showed higher activity with TCP as compared to wild-type, and further mutations enhanced the stability of the enzymes.

The strategy to construct a recombinant TCP-degrading strain was based on the use of an improved haloalkane dehalogenase into an organism that grows on the product of hydrolytic dehalogenation, which is 2,3-dichloro-1-propanol. For this, a host was used that could degrade both 2,3-dichloropropanol and 1,3-dichloropropanol: *Agrobacterium radiobacter* AD1 (van den Wijngaard et al. 1989). First, the wild-type haloalkane dehalogenase gene for DhaA from *Rhodococcus* was placed under control of a strong constitutive promoter and cloned on a broad host range plasmid (pLAFR3) that was introduced into strain AD1 (Bosma et al. 1999). Growth of the resulting strain was not significant, but after incubation of 25 days 0.7 mM of TCP was converted and a small increase of biomass was observed. The strain did utilize 1,2,3-tribromopropane and 1,2-dibromo-3-chloropropane as sole carbon source, showing for the first time growth on a trihalopropane.

Growth on TCP could be obtained when a DhaA-type dehalogenase with improved activity for TCP was used. The *dhaAM2* gene for the improved dehalogenase was constitutively expressed in strain AD1. The resulting strain, *A. radiobacter* AD1(pTB3-M2), was able to utilize TCP as carbon and energy source under aerobic conditions. After 10 days, 3.6 mM TCP was converted by a culture initially inoculated to an OD_450_ of 0.14. Due to production of hydrochloric acid, the pH dropped to 6.0 (Bosma et al. 2002).

The construction of a recombinant strain using an improved haloalkane dehalogenase that was expressed under a strong constitutive promoter in a host that degrades a dichloropropanol, is an important step towards obtaining an organism that is suitable for TCP bioremediation under aerobic conditions. However, the system has still limitations and drawbacks (Bosma et al. 2002): (1) Although the initial dehalogenase is significantly improved for TCP conversion (ca. 5-fold as compared to wild-type), the activity of DhaAM2 is still too low to rapidly transform TCP. Consequently, the estimated doubling time of the constructed strain was 90 h, which, for comparison, is much slower than the ca. 10 h measured for the 1,2-dichloroethane-degrader *Xanthobacter autotrophicus* strain that is used for full-scale groundwater bioremediation. (2) Degradation of TCP was incomplete due to the enantioselective conversion of only the (*R*)-2,3-dichloropropanol by the host *A. radiobacter* AD1. The DhaAM2 dehalogenase produced a racemic mixture of (*R*)- and (*S*)-2,3-dichloropropanol from TCP. (3) The modified dehalogenase gene for DhaAM2 was introduced into strain AD1 using the cloning vector pLAFR3, which is a transmissible plasmid. Such a plasmid may be modified or lost under stress conditions, or it may be transferred to other bacteria. (4) Application of specialized bacteria in bioremediation operations will likely make use of open systems, such as an immobilized-cells bioreactor from which organisms may detach and end up in effluent water. This may lead to spread of resistance genes (in this case tetracycline) if the engineered organism contains additional antibiotic resistance markers. To remedy these limitations, further improvements are under investigation.

### Prospects

The catabolic potential of naturally occurring organisms towards organic compounds is the result of long evolution processes, whereas the time in which organisms have been tempted to evolve new enzymes, pathways and regulatory mechanisms that allow conversion of xenobiotic industrial chemicals is quite short. The industrial synthesis of compounds such as trichloropropane only started in the first half of the 20th century. Nevertheless, the presence of these synthetic compounds in the biosphere has already triggered the evolution of new metabolic activities, as illustrated by various examples (Janssen 2004; Janssen et al. 2005; Paul et al. 2005).

An important example of bacteria capable of TCP degradation are the strictly anaerobic strains BL-DC-8 and BL-DC-9 of *D. lykanthropropepellens*, isolated from contaminated groundwater in the USA (Yan et al. 2009). The net dihaloelimination reaction catalyzed by these organisms implies transfer of electrons to TCP, with chloride release. This suggests the possibility of reductive dehalogenation coupled to electron transfer from hydrogen or another electron donor to TCP (dehalorespiration; Smidt and de Vos 2004). Since this process could possibly stimulate growth, as indicated by an increase in cell numbers (Yan et al. 2009), genetic- or population-level adaptation of cultures to TCP under anaerobic conditions can be envisaged. This may yield faster growing cultures than those currently described (maximum specific growth rate 0.15–0.17 day^−1^). It would also be highly interesting to identify the genes, proteins and cofactors involved in anaerobic conversion of TCP to allyl chloride and to establish their possible association with energy metabolism. The biochemical basis of dihaloelimination reactions is currently not well understood, although they may be important for different chlorinated substrates (de Wildeman et al. 2003; Smidt and de Vos 2004). For in situ bioremediation, anaerobic transformation may be more attractive than aerobic processes, due to the difficulty of homogeneous oxygen supply and its preferred use for other oxidative processes if TCP is a low-level contaminant.

Anaerobic degradation of TCP was described to produce next to allyl chloride also small amounts of further conjugation products (diallyl sulfide, allyl mercaptan), probably due to abiotic reactions with sulfide (Yan et al. 2009). The chemically labile carbon–halogen bond in allyl chloride, as well as its sensitivity to cleavage by hydrolytic dehalogenases, suggest that more rapid biodegradation of allyl chloride with reduced formation of sulfur conjugates can be achieved when adapted mixed cultures are used. Thus, further studies on the anaerobic metabolism of TCP and allyl chloride, in combination with appropriate enrichment and adaptation strategies, may well lead to more rapid anaerobic degradation as compared to what is currently possible.

Regarding aerobic degradation of TCP, genetic engineering can contribute to the acquisition of new bioremediation organisms, as illustrated by Bosma et al. (2002). To further enhance the biodegradation of TCP, use of a better haloalkane dehalogenase is desirable. By using rational design and directed evolution, the activity of DhaA against TCP was recently improved by Pavlova et al. (2009). Tunnel residues leading to the active site of DhaA were selected as target spots for mutagenesis, based on the notion that substrate binding and/or product release may limit the rate of catalysis. The best variants that were obtained carried three new mutations as compared to variant DhaAM2, and had 36 times higher activity (k_cat_) than the natural enzyme towards TCP (Table 1). In the degradation pathway of 1,2-dichloroethane (DCE) by *X. autotrophicus* GJ10, the first step is catalyzed by DhlA, which is a phylogenetically related haloalkane dehalogenase. Since this organism was successfully used for groundwater cleanup at full scale (Stucki and Thüer 1995), it is interesting to compare the catalytic rates of the initial haloalkane dehalogenases (Table 1). The differences in Table 1 are important since kinetic properties and expression levels of the dehalogenases have a major impact on the kinetic properties of chloroalkane degradation (substrate affinity, growth rate) by the host organism (van den Wijngaard et al. 1993). Even though the activity of DhaA31 is significantly improved by directed evolution, the *k*_cat_ and *k*_cat_/*K*_m_ values of DhaA31 for TCP are still lower than the corresponding values of DhlA for DCE (Table 1). Thus, an engineered organism expressing the evolved DhaA31 will still have a lower affinity for TCP than strain GJ10 for DCE. It is well possible that further variants of haloalkane dehalogenases that convert TCP even better can be obtained. Strategies for laboratory evolution of new enzyme activities are still improving, and recently we were able to obtain complementary TCP dehalogenating mutants that produce almost enantiopure (*R*)- or (*S*)-2,3-dichloro-1-propanol. Although dehalogenase enantioselectivity may be unimportant for groundwater and soil bioremediation, it holds great promise for converting TCP waste to economically valuable chiral building blocks for use in the fine chemicals and pharmaceutical industries (van Leeuwen et al. 2012).

**Table 1:** Kinetic parameters of haloalkane dehalogenase variants with TCP and 1,2-dichloroethane (DCE)

Variant	Substrate	*k*_cat_ (s^−1^)	*K*_m_ (mM)	*k*_cat_/*K*_m_ (M^−1^ s^−1^)	Reference
DhaA wild-type	TCP	0.035 ± 0.002	0.98 ± 0.17	36	Bosma et al. (2002)
DhaAM2	TCP	0.28^a^	1.0^a^	280	Bosma et al. (2002)
DhaA27	TCP	1.02 ± 0.031	1.09 ± 0.10	930	Pavlova et al. (2009)
DhaA31	TCP	1.26 ± 0.031	1.2 ± 0.15	1060	Pavlova et al. (2009)
DhlA wild-type	DCE	3.3 ± 0.5	0.53 ± 0.2	6200	Krooshof et al. (1998)

DhaA and variants thereof indicate the *Rhodococcus* enzyme that was subjected to directed evolution for enhanced TCP conversion. DhlA indicated the *X. autotrophicus* dehalogenase that was applied in a whole-cell cleanup process for 1,2-dichloroethane removal

^a^Margin of error not given

Improved conversion of 2,3-dichloropropanol by a better host is under investigation with new isolates that were obtained from a site contaminated with epichlorohydrin and chloropropanols due to leakage of waste from epichlorohydrin manufacture. This organism, a strain of *Pseudomonas putida*, uses a pathway for 2,3-dichloropropanol degradation that is different from the route detected in *Agrobacterium* strains (Higgins et al. 2005; van den Wijngaard et al. 1989) and lacks enantioselectivity. However, none of the current dichloropropanol degraders has been selected on the basis of its potential to form a biofilm on a solid support under groundwater flow conditions, and in competition with other bacteria. Furthermore, substrate supply will likely be low, which also may impose physiological requirements on the host organism.

The use of plasmid-based systems, as in the *A. radiobacter* AD1(pTB3-M2) recombinant (Bosma et al. 2002) is undesirable for the construction of bioremediation organisms, especially when in situ remediation is targeted (de Lorenzo and Timmis 1994; Timmis and Pieper 1999). A recombinant organism applied in situ should be capable of establishing itself an environment where the conditions cannot be controlled (de Lorenzo 2009). This may cause stress, leading to plasmid loss or lysis, as well as to spread of recombinant DNA. The presence of antibiotic resistance-based selection markers and the use of transmissible plasmids can be avoided by employing chromosomal integration, for which efficient transposon-based systems were developed. For example, a modified Tn5 transposon system can be used to integrate a foreign gene into the chromosome, leading to stable integration (de Lorenzo and Timmis 1994). Such cloning vectors have been used successfully to construct strains for environmental applications (Panke et al. 1998).

If an efficient pathway can be assembled or evolved in the laboratory, in a robust host organism that can maintain itself under practical conditions, the prospects of successful application of such a genetically engineered organism for bioremediation are good. The limited success that has been achieved so far in this area, is mainly due to the fact that few recombinant organism have been engineered to degrade compounds which are really recalcitrant and where the poor degradability is due to biochemical factors instead of low solubility, limiting oxygen supply, poor bioavailability, etc. On the other hand, evolution of dehalogenases also occurs in natural environments (Janssen 2004), and it is well possible that at some day, due to continued evolutionary pressure, TCP becomes a degradable compound and that TCP-degrading organisms can be obtained by classical enrichment.

## Conclusions

The toxicity and environmental behavior of TCP has stimulated research into techniques for removal of TCP from polluted sites. However, cleanup of TCP-contaminated water and soil is difficult due to its physiochemical properties and persistent nature. Biodegradation could be an attractive approach if suitable cultures become available.

Both aerobic and anaerobic processes have been investigated, but further work is needed to obtain cultures and processes with sufficient activity for testing under practical conditions and scale-up. Until then, water treatment can be done by chemical methods such as oxidative degradation using a strong oxidant and a catalyst or UV light to generate radicals. Reductive dechlorination by zero-valent iron and especially zinc are also suitable options, also for in situ application as barriers to prevent spreading via groundwater flow. Soil venting, stripping and activated carbon absorption may be used for removing TCP contaminants from soil and water. For in situ treatment, reductive dechlorination may be the best option, especially if it can be coupled to growth-supporting dehaloelimination.

Recent developments in molecular biology and protein engineering have led to the construction of genetically engineered strains that allow slow but complete biodegradation of TCP under aerobic conditions. If these strains can be further evolved to exhibit degradation rates that compare favorably or are similar to those of 1,2-dichloroethane degradation by *X. autotrophicus* GJ10, which is used at full scale for groundwater cleanup (Stucki and Thüer 1995), it is likely that a full-scale TCP bioremediation is feasible. The physico-chemical properties of the two compounds are very similar. The construction of such strains is dependent on dehalogenase with high activity, robust host strains that resist uncontrollable conditions, and the possibility to obtain growth-supporting metabolic pathway that completely mineralizes TCP.

## Abbreviations

TCP: 1,2,3-Trichloropropane
